# Supplementary material for: Prevalence and factors associated with peripheral neuropathy in a setting of retail pharmacies in Malaysia–A cross-sectional study
Source: PLoS One. 2024 Oct 29;19(10):e0307093. doi: 10.1371/journal.pone.0307093 (PMC11521241; doi:10.1371/journal.pone.0307093)
Supplement: S1 Table — (DOCX) [file pone.0307093.s001.docx]

S1 Table: Neuropathy symptom score

| Variables | | Score |
| --- | --- | --- |
| 1 | Please tick one answer  □ Burning/numbness/tingling ( 2 marks)  □ Fatigue/cramping/aching ( 1 mark)  □ no symptoms ( 0 mark) |  |
| 2 | Please tick one answer  □ Symptoms present in feet ( 2 mark)  □ Symptoms present in calves ( 1 mark)  □ Symptoms present elsewhere ( 2 mark) |  |
| 3 | Please tick one answer  □ Nocturnal exacerbation of symptom ( 2 mark)  □ Symptoms present day and night ( 1 mark)  □ Symptoms present at daytime only ( 0 mark) |  |
| 4 | Please tick if applicable to you  □ Symptoms wake you up from sleep ( 1 mark) |  |
| 5 | Please tick one answer,  manoeuvres to reduce symptoms:  □ walking ( 2 mark)  □ standing ( 1 mark)  □ sitting/lying ( 0 mark) |  |
| Note: the score could range from 0-9. Severity of peripheral neuropathy can be categorized into Normal (sum of score 0-2), mild symptom (sum of score of 3-4), moderate symptoms (sum of score 5-6) and severe symptoms (sum of score 7-9). | | |
