# Supplementary material for: Prevalence and factors associated with peripheral neuropathy in a setting of retail pharmacies in Malaysia–A cross-sectional study
Source: PLoS One. 2024 Oct 29;19(10):e0307093. doi: 10.1371/journal.pone.0307093 (PMC11521241; doi:10.1371/journal.pone.0307093)
Supplement: S2 Table — (DOCX) [file pone.0307093.s002.docx]

|  | | Right | Left |
| --- | --- | --- | --- |
| 1 | Ankle reflex  □ Normal (0 mark)  □ With reinforcement (1 mark)  □ Absent (2 mark) |  |  |
| 2 | Vibration perception  □ Normal (0 mark)  □ Reduced/Absence (1 mark) |  |  |
| 3 | Pin prick sensation  □ Present (0 mark)  □ Reduced/absence (1 mark) |  |  |
| 4 | Temperature  □ Present (0 mark)  □ Reduced (1 mark) |  |  |
| Note: the score could range from 0-10. Normal (sum of score 0-2), mild sign of peripheral neuropathy (sum of score 3-5), moderate sign (sum of score 6-8), and severe sign (sum of score 9-10). | | | |

S2 Table: Neuropathy disability score
