# Supplementary material for: Prevalence and factors associated with peripheral neuropathy in a setting of retail pharmacies in Malaysia–A cross-sectional study
Source: PLoS One. 2024 Oct 29;19(10):e0307093. doi: 10.1371/journal.pone.0307093 (PMC11521241; doi:10.1371/journal.pone.0307093)
Supplement: Attachment — Submitted filename: RV Per Neuropathy paper_Guidelines for Reviewers PlosOne 10.2023.docx [file pone.0307093.s003.docx]

Line 100

Comparison between previous studies, in reference 12-13 are from years 2003-2012. Is there any newer study?

Reference 15 is on painful neuropathy from 2017, showing a lesser prevalence than references 12-13; is there any newer study? If not, perhaps can mention, “in the absence of other studies, the latest prevalence was... in year ... “

Line 108

Is there any reference for poor awareness among patients and less accessibility to gain proper attention from physicians? How about PHC? In Malaysia, PHC teams have been trained to identify DM wounds and have dedicated DM clinics and wound care teams. So, should be careful with these kinds of strong statements. It seems to pinpoint the reason for DM wounds is due to lack of access to treatment.

Line 125 Methods

How come the sample is taken as those aged more than 18? Most diabetics are older.

Where was this convenient sampling done? Only later is it mentioned as a single location. Why was it not done in more locations? Is there data to support that by taking a sample from one location, it can be generalised to all over Malaysia? Can at least mention which location it was.

Line 132 sample size calculation

Any justification on similarity of Parsis in Mumbai/Bombay and Malaysia?

Line 150 biothesiometer usage, line 166 examination

What if they had a wound/ ulcer on the foot? Were they excluded?

Line 183 Statistical analysis

How about other comorbids eg hypercholesterolemia /IHD?

The outcome variable, severity of peripheral neuropathy is only explained in next section (line 194, operational definition), whereas here under statistical analysis, scoring here and in methods was for neuro symptoms and neuro disability. Perhaps could be made a little clearer.

Line 212 baseline characteristics

If we are asking about diet (nonveg), how about hyperlipidemia? Especially if can ask about DM and HPT (self-reported).

Line 305 Limitations

single location- have mentioned as above

Line 331

Retail pharmacists or pharmacies?

Line 337

Good public health implications and inferences.

Line 475 tables

Perhaps can rewrite min – max as min to max, so as not to denote a mathematical equation.

Ethnicity, how come Chinese are highest? Whereas Malays are the largest ethnic group? Is there any reason that can explain this finding. If so, should elaborate in the discussion.
