# Supplementary material for: Prevalence and factors associated with peripheral neuropathy in a setting of retail pharmacies in Malaysia–A cross-sectional study
Source: PLoS One. 2024 Oct 29;19(10):e0307093. doi: 10.1371/journal.pone.0307093 (PMC11521241; doi:10.1371/journal.pone.0307093)
Supplement: Attachment — Submitted filename: Authors comments.docx [file pone.0307093.s004.docx]

Thank you for the comments to improvise the manuscript, we have revised the manuscript based on the comments provided and used the track changes for revision.

**Reviewer Comments**

1. Line 100 - Comparison between previous studies, in reference 12-13 are from years 2003-2012. Is there any newer study? Reference 15 is on painful neuropathy from 2017, showing a lesser prevalence than references 12-13; is there any newer study? If not, perhaps can mention, “in the absence of other studies, the latest prevalence was... in year ... “

Author response: We found two meta-analysis report prevalence of DPN among T1DM, T2DM and pre-diabetes. We have inserted the two reviews in the paragraph, to read it as:

“Overall, the finding from a recent meta-analysis showed that the pooled prevalence of DPN was 30% and DPN is more prevalent in people with T2DM (31.5%) compared to those in T1DM (17.5%) [15]. Prevalence of peripheral neuropathy in patients with diabetes: A systematic review and meta-analysis. Primary care diabetes, 14(5), 435-444). Another meta-analysis also report that the DPN was also common (>10%) among those with pre-diabetes [16].

**Reviewer Comments**

2. Line 108 - Is there any reference for poor awareness among patients and less accessibility to gain proper attention from physicians? How about PHC? In Malaysia, PHC teams have been trained to identify DM wounds and have dedicated DM clinics and wound care teams. So, should be careful with these kinds of strong statements. It seems to pinpoint the reason for DM wounds is due to lack of access to treatment.

Author response: Sorry for the confusion, we have replaced that sentence with another one, to read it as:

“~~The diagnosis of peripheral neuropathy has been overlooked especially among the general population, due to poor awareness among patients and less accessibility to gain proper attention from physicians for early symptoms presentation~~. Lack of widespread awareness about peripheral neuropathy, coupled with its subtle symptoms that don't significantly disrupt daily life, often results in individuals with this condition neglecting to seek medical attention for these minor manifestations.”

**Reviewer Comments**

3. Line 125 Methods - How come the sample is taken as those aged more than 18? Most diabetics are older. Where was this convenient sampling done? Only later is it mentioned as a single location. Why was it not done in more locations? Is there data to support that by taking a sample from one location, it can be generalised to all over Malaysia? Can at least mention which location it was.

Author response – This was a community study involving 7 retail pharmacies. (refer Study design, study population and sampling method). The objective of this study was to establish the prevalence of peripheral neuropathy among the general population. Therefore, we included those aged ≥18 years old with convenient sampling method, regardless of diabetes status. The community outreach's purpose is to increase awareness, not just focusing on peripheral neuropathy among people with diabetes. We have made necessary amendment in limitation section, to read it as:

“This study must be interpreted with a few limitations. First, the study had a cross-sectional design, which means that it was conducted at a single point in time, and the researchers could not examine the causality between the predictors and the outcome. Second, the participants were recruited from ~~a single~~ ~~location~~ retailed pharmacies, which may limit the generalizability of the findings to other populations or settings. Third, the study relied on self-reported data, which may be subject to bias, as participants may not have accurately reported their medical history or symptoms. Fourth, the study did not capture all potentially relevant variables, such as BMI, pre-diabetes status, and prior diagnosis of peripheral neuropathy, which may limit the accuracy and precision of the findings.”

**Reviewer Comments**

4. Line 132 sample size calculation - Any justification on similarity of Parsis in Mumbai/Bombay and Malaysia?

Author response: I agree with you that there could be no similarity between Parsis (Mumbai) and Malaysian population, but that study (20.Bharucha NE, Bharucha AE, Bharucha EP. Prevalence of peripheral neuropathy in the Parsi community of Bombay. Neurology. 1991 Aug 1;41(8):1315-.) is the only community study involved general public. Therefore we used it as the reference for sample size calculation. In addition, For this study, a non-probability sampling method was employed. The application of traditional sample size calculations in such scenarios aimed to ensure a sufficient amount of data, yielding reasonable precision and meaningful outputs within the study's constraints. Sample size calculation was more crucial to a study conducted with a probability sampling method.

**Reviewer Comments**

5. Line 150 - biothesiometer usage, line 166 examination. What if they had a wound/ ulcer on the foot? Were they excluded?

Author response – Exclusion was not applied to individuals with foot ulceration or wounds. The data pertaining to "Diabetic-related complications among individuals with diabetes" had been shown in Table 1.

**Reviewer Comments**

6. Line 183 Statistical analysis - How about other co-morbids eg hypercholesterolemia /IHD?

Author response –

In response to the inquiry about including other comorbidities like hypercholesterolemia and ischemic heart disease (IHD), we value the reviewer's suggestion. However, it's important to note that our data collection sheet did not encompass these specific comorbidities. Our primary focus was on variables directly pertinent to the study's objective, which is to investigate the prevalence and factors associated with peripheral neuropathy in the community level.

Even though we had collected the diabetic-related complications like IHD but the sample size was small (n=1) as shown in Table 1. Nevertheless, we acknowledge the merit of a more comprehensive approach in future research endeavors. We appreciate the suggestion and plan to consider the incorporation of additional comorbidities, including hypercholesterolemia and IHD, to enrich the depth of our analysis. This approach will contribute to a more thorough examination of potential associations in our future studies.

**Reviewer Comments**

7. The outcome variable, severity of peripheral neuropathy is only explained in next section (line 194, operational definition), whereas here under statistical analysis, scoring here and in methods was for neuro symptoms and neuro disability. Perhaps could be made a little clearer.

Author response – Sorry for the confusion, we have amended the sentence, to read it as:

The dependent variable, categorized as "yes" in the presence of mild to severe symptoms of peripheral neuropathy, underwent testing through either the Chi-Square test or Fisher’s exact test. Variables with a p-value < 0.25 in the simple logistic regression (derived from the results in the normal versus mild-severe column) were entered into multiple logistic regressions to look for determinants of peripheral neuropathy (Mild-severe). The level of significance is set at a p-value < 0.05.

**Reviewer Comments**

8. Line 212 baseline characteristics. If we are asking about diet (nonveg), how about hyperlipidemia? Especially if can ask about DM and HPT (self-reported).

Author response - We acknowledge the reviewer's suggestion to include hyperlipidemia in the baseline characteristics, particularly in relation to inquiries about a nonvegetarian diet. While we collected self-reported information on diabetes (DM) and hypertension (HPT) due to their association with peripheral neuropathy, hyperlipidemia was not included in the data collection sheet as the correlation between statin therapy and peripheral neuropathy is a matter of debate.(reference: Pasha R, Azmi S, Ferdousi M, Kalteniece A, Bashir B, Gouni-Berthold I, Malik RA, Soran H. Lipids, Lipid-Lowering Therapy, and Neuropathy: A Narrative Review. Clin Ther. 2022 Jul;44(7):1012-1025. doi: 10.1016/j.clinthera.2022.03.013. Epub 2022 Jul 6. PMID: 35810030.

However, we acknowledge the significance of assessing hyperlipidemia in relation to dietary habits and will consider including this variable in future research to provide a more comprehensive understanding of baseline characteristics.

**Reviewer Comments**

9. Line 305 Limitations - single location- have mentioned as above

Author response – We have amended the typo, to read it as:

“This study must be interpreted with a few limitations. First, the study had a cross-sectional design, which means that it was conducted at a single point in time, and the researchers could not examine the causality between the predictors and the outcome. Second, the participants were recruited from ~~a single~~ ~~location~~ retailed pharmacies, which may limit the generalizability of the findings to other populations or settings. Third, the study relied on self-reported data, which may be subject to bias, as participants may not have accurately reported their medical history or symptoms. Fourth, the study did not capture all potentially relevant variables, such as BMI, pre-diabetes status, and prior diagnosis of peripheral neuropathy, which may limit the accuracy and precision of the findings.”

**Reviewer Comments**

10. Line 331 - Retail pharmacists or pharmacies?

Author response – We have amended the typo, to read it as:

“To our knowledge, this is the first comprehensive study among the prevalence and the factors of peripheral neuropathy among retail ~~pharmacists~~ pharmacies in Malaysia using a biothesiometer.”

**Reviewer Comments**

11. Line 337 - Good public health implications and inferences.

Author response – Thank you for your enlightenment, we have strengthened the implications with the following changes, to read it as:

“By identifying the underlying mechanisms and risk factors associated with this condition, ~~researchers can develop more effective treatments and preventative measures to improve the quality of life for those affected by peripheral neuropathy~~ this study signifies that there is a need for targeted public health campaigns aimed at increasing awareness about the condition, its risk factors, and early symptoms, which encompass diverse ethnic groups and emphasize the importance of regular health check-ups, particularly for individuals with comorbidities such as diabetes and hypertension. Furthermore, healthcare providers play a crucial role in public awareness, such as training programs for healthcare professionals could emphasize the significance of early detection, prompt intervention, and patient education regarding peripheral neuropathy. This, in turn, can contribute to improved patient outcomes and a reduced burden on healthcare systems.”

**Reviewer Comments**

12. Line 475 tables - Perhaps can rewrite min – max as min to max, so as not to denote a mathematical equation.

Respond: Amendment done.

**Reviewer Comments**

13. Line 475 Table:Ethnicity, how come Chinese are highest? Whereas Malays are the largest ethnic group? Is there any reason that can explain this finding. If so, should elaborate in the discussion.

Author response – We have addressed this finding under limitation (line 332) to read as:

The Malay ethnic group constitutes the majority in Malaysia, but in this study, Chinese respondents accounted for 54.1%. This discrepancy may be attributed to a higher prevalence of Chinese clients at the selected retail pharmacies and their increased interest in assessing their peripheral neuropathy status. It's important to note that the distribution of ethnic groups in this study does not accurately reflect the actual demographic proportions in Malaysia; rather, it is a consequence of employing a non-probability sampling method.
