# Supplementary material for: Prevalence and factors associated with peripheral neuropathy in a setting of retail pharmacies in Malaysia–A cross-sectional study
Source: PLoS One. 2024 Oct 29;19(10):e0307093. doi: 10.1371/journal.pone.0307093 (PMC11521241; doi:10.1371/journal.pone.0307093)
Supplement: Attachment — Submitted filename: Response to the reviewers.docx [file pone.0307093.s005.docx]

Journal Requirements:

Please review your reference list to ensure that it is complete and correct. If you have cited papers that have been retracted, please include the rationale for doing so in the manuscript text, or remove these references and replace them with relevant current references. Any changes to the reference list should be mentioned in the rebuttal letter that accompanies your revised manuscript. If you need to cite a retracted article, indicate the article’s retracted status in the References list and also include a citation and full reference for the retraction notice.

Answer: We have made the changes accordingly to all the references

Review Comments to the Author

**Reviewer #1:** The authors have mostly addressed the queries put forth earlier. Just a few to improve the manuscript before publication.

Minor comments on:

Comment 1

Intro Line 98: Universiti Hospital – should this be spelt University Hospital, or University Malaya Medical Centre? If it is not UMMC, then should clearly identify which hospital this is, without reader having to view the reference.

Answer: We have made the changes to read as” University Kebangsaan Malaysia Medical Center” (page 4, line 96)

Comment 2

Intro line 101: USM – pls write in full

Answer: We have revised the sentences (page 5, line 101)

Comment 3

Intro overall seems to focus on DM neuropathy. What about other types of neuropathy?

Answer: Thank you for your input. We acknowledge that there are other types of neuropathy, such as Guillain-Barré syndrome (GBS), chronic inflammatory demyelinating polyneuropathy (CIDP), amyloid neuropathy, and acute inflammatory neuropathies. However, given the high prevalence of diabetes in Malaysia, diabetic neuropathy is likely the most common form of neuropathy in the general population, making it a significant early complication of diabetes. Therefore, our focus remains on diabetic neuropathy due to its prevalence and the potential for early detection.

We have added the following paragraph in the main text, to read it as: (page 5, line 115 to 126)

Diabetes mellitus is a highly prevalent condition in the Malaysian population, and diabetic neuropathy is one of the earliest and most common complications arising from it. As a large segment of the population is affected by diabetes, it is reasonable to assume that diabetic neuropathy is likely the most common form of neuropathy encountered in the general population. In contrast, other types of neuropathies mentioned, such as Guillain-Barré syndrome (GBS), chronic inflammatory demyelinating polyneuropathy (CIDP), amyloid neuropathy, and acute inflammatory neuropathies, are relatively rare and often present with rapidly progressive or severe symptoms that would prompt individuals to seek medical attention promptly. These forms of neuropathy are typically more severe and require immediate medical intervention, making them less relevant for the specific focus of this paper, which primarily addresses early detection and monitoring of neuropathy in the general population [15,16,17].

Comment 4: Sample size calculation lines 133-137: should add what you had explained in the rebuttal to my earlier comment in the first review about why you are comparing/ referring to a study conducted among the Parsi community in Mumbai, India, so that the reader understands your rationale as well.

Answer: We have added this sentence to read as (page 7, line 165-171)

While determining the appropriate sample size for our study on the prevalence of diabetic neuropathy in the community, we encountered a scarcity of recent, locally relevant research to serve as a reference. The paucity of community-based neuropathy studies, particularly in our region, necessitated the use of an older study conducted in Mumbai, India, in 1991 [22]. Despite its age, this study remains one of the few comprehensive investigations into the prevalence of neuropathy in a community setting, making it a valuable resource for our sample size calculations.

Comment 5: This integration maintains the focus of your study while explaining the rationale behind your methodological choices.

We have added this in the main text to read as: (page 6-7; line 144-155)

Retail pharmacies were selected as data collection sites based on several considerations. Firstly, individuals with chronic diseases, such as diabetes, frequently visit retail pharmacies to obtain their medications or seek alternative therapeutic options like dietary supplements. These individuals are already engaged with pharmacists in managing their health conditions. By conducting this study at retail pharmacies, we could access a significant portion of the target population who are likely to be more health-literate and inclined to learn about their health status.

Additionally, we conducted this study in the form of a health campaign offering free neuropathy screenings, which further facilitated convenient data collection. This approach not only benefits those with diabetes by providing them with an opportunity to assess their neuropathy status, but also allows individuals without diabetes to undergo screening and increase their awareness about neuropathy. Furthermore, the health campaign raised awareness among the public visiting the pharmacy, including family members or companions of those with diabetes.

Comment 6: Discussion line 273 grammatical correction, should read as, “The higher prevalence of peripheral neuropathy in our study population compared to global studies could be attributed to the biothesiometer's higher sensitivity in detecting early or mild peripheral neuropathy, as well as
the possibility of many Malaysians having undiagnosed diabetes mellitus, on par with the increasing obesity epidemic in Malaysia.”

Answer: We have amended the paragraph as suggested page 13, line 303 to 307.

Comment 7: Discussion line 278- since you mention other ethnicities as significant for those aged >50, I feel you should substantiate the majority of Chinese ethnic respondents, as mentioned in your rebuttal to myfirst review of your draft. It is clearly mentioned there, so do incorporate the baseline discussion of your findings in your paragraph, or at least why the majority are Chinese here.

Answer: Thank you for your observation. The majority of Chinese ethnic respondents in retail pharmacies can be attributed to several factors. In Malaysia, Chinese Malaysians represent a significant proportion of the urban population, where retail pharmacies are predominantly located. Additionally, cultural and socioeconomic factors may contribute to a higher propensity among the Chinese community to seek health services from retail pharmacies.

We have added the following explanation to the discussion: (page15, line 351-362)

The Malay ethnic group constitutes the majority in Malaysia, but in this study, Chinese respondents accounted for 54.1%. The predominance of Chinese ethnic respondents in our study can be attributed to several factors. Chinese Malaysians constitute a significant segment of the urban population, where retail pharmacies are mainly concentrated. Furthermore, cultural attitudes and socioeconomic status may influence the likelihood of individuals within the Chinese community to seek health services from retail pharmacies. This tendency could be related to greater health awareness, accessibility, and a preference for retail pharmacies for minor health concerns among this ethnic group. It is important to note that the distribution of ethnic groups in this study does not accurately reflect the actual demographic proportions in Malaysia; rather, it is a consequence of employing a non-probability sampling method. These limitations should be considered when interpreting the findings of this study, and future studies should aim to address these limitations.

Comment 8: Strengths and limitations line 309: when you mention single location, do elaborate- do you mean single state/ single region? Sounds better than one location alone which sounds like the researchers did not put in more effort, whereas you have actually gone to 7 different pharmacies.

Answer: That was referring to single state with seven retail pharmacies. We have revised the sentence to read as (page 14, line 340-343 ):

This study must be interpreted with a few limitations. First, the study had a cross-sectional design, and the researchers could not examine the causality between the predictors and the outcome. Second, participants were recruited from seven retail pharmacies located in the state of Selangor, which may limit the generalizability of the findings to other populations or settings.

Comment 9: Line 332 – I think you mean retail pharmacies, otherwise it would mean you are studying the retail pharmacists as your sample.

Answer: yes. You are right. Correction had been made.

Comment 10: Line 376 – the findings presented in the results (add the s)

Answer: We have made the amendment as suggested (Line 376)

Comment 11: Conclusion line 394 – to prevent associated complications.

Answer: We have made the amendment as suggested (Line 394)

**Reviewer #2:**

**Comment 1:** I recommend adjusting the title and add the word factors instead of factor.

Answer: We have amended the title accordingly to read as

“Prevalence and factors associated with peripheral neuropathy in a setting of retail pharmacies in Malaysia – A cross-sectional study”

Comment 2: The sample size calculation isn't accurate.

Answer: We acknowledge that the optimal approach for calculating the sample size would have been based on the two-proportion formula, considering the current study aims to identify factors associated with peripheral neuropathy. The initial sample size was calculated using the StatCalc function in Epi Info 7.0, based on the prevalence of peripheral neuropathy among the Parsi community of Bombay, which was 2.38% [22]. This calculation resulted in an estimated sample size of 899, with a 95% confidence interval (CI), 80% power, and a p-value of <0.05. After accounting for a potential missing value rate of 30%, the total required number of participants was determined to be 1,283.

We did not use the prevalence of peripheral neuropathy from studies conducted at UKM, UM, or USM, as these studies were among diabetic patients in outpatient clinics. This setting could lead to selection bias, as patients at outpatient clinics are more likely to have advanced or severe forms of the condition, resulting in an overestimation of prevalence. Such overestimation could inflate the sample size requirement, not accurately reflecting the true population parameters. Therefore, we adhered to the original reference for sample size calculation, which was the Mumbai community study. My study aims to assess the prevalence and associated factors of peripheral neuropathy in a community setting, similar to the Mumbai study. Using a reference study with a comparable population and setting ensures greater alignment with the research objectives and enhances the validity of the sample size calculation.

For the factors associated with peripheral neuropathy, we used G*Power software, which calculated a required sample size of 8,422. This calculation was based on a study conducted at Universiti Kebangsaan Malaysia, where older age was identified as a determinant of diabetic peripheral neuropathy among patients with diabetes (OR 1.13, 95% CI 1.01-1.26, p=0.039) [22]. Due to the impractically large sample size and time constraints, we adopted a sample size of 899 for this study.

Reference: Faul, F., Erdfelder, E., Lang, A.-G., & Buchner, A. (2007). G*Power 3: A flexible statistical power analysis program for the social, behavioral, and biomedical sciences. Behavior Research Methods, 39, 175-191.).

We have added statement under sample size calculation session (line 172-177)

For the factors associated with peripheral neuropathy, we used G*Power software, which calculated a required sample size of 8,422. This calculation was based on a study conducted at Universiti Kebangsaan Malaysia, in which older age was identified as a determinant of diabetic peripheral neuropathy among patients with diabetes (OR 1.13, 95% CI 1.01-1.26, p=0.039) [13]. Due to the impractically large sample size and time constraints, we adopted a sample size of 899 for this study.

Comment 3: The tables need to be more justified.

Answer: We have simplified the tables now.

Table 1: Characteristics of participants’ socio-demographics, lifestyle, medical background, family history, and diabetic-related complications (n=1283)-remained

Table 2: Percentage of peripheral neuropathy based on different tests still remained

Table 3 on Percentage of peripheral neuropathy (screened by biothesiometer) according to age groups have been deleted from the main text

Table 4 becomes Table 3 and the presentation has been simplified it as follow:

Table 3: Factors associated with severity of peripheral neuropathy using chi-square (n=1283)

| Variables | Category | Normal, 943 (73.5) | Mild-severe, 340 (26.5) | P-values |
| --- | --- | --- | --- | --- |
| Age | ≤50 | 655 (87.4) | 94 (12.6) | <0.001 |
|  | >50 | 288 (53.9) | 246 (46.1) |  |
| Gender | Male | 448 (69.1) | 200 (30.9) | <0.001 |
|  | Female | 495 (78.0) | 140 (22.0) |  |
| Ethnicity | Malay | 302 (81.2) | 70 (18.8) | 0.001 |
|  | Chinese | 492 (70.9) | 202 (29.1) |  |
|  | Indian | 111 (68.9) | 50 (31.1) |  |
|  | Others | 38 (67.9) | 18 (32.1) |  |
| Education | None | 16 (41.0) | 240 (39.3) | <0.001 |
|  | Primary | 54 (42.9) |  |  |
|  | Secondary | 301 (67.5) |  |  |
|  | Pre-U | 96 (83.5) | 100 (14.9) |  |
|  | Tertiary | 476 (85.5) |  |  |
| Drink Alcohol | No | 757 (73.2) | 277 (26.8) | 0.633 |
|  | Yes | 186 (74.7) | 63 (25.3) |  |
| Smoking | No | 802 (74.9) | 269 (25.1) | 0.012 |
|  | Yes | 141 (66.5) | 71 (33.5) |  |
| Vegetarian | No | 923 (73.7) | 329 (26.3) | 0.251 |
|  | Yes | 20 (64.5) | 11 (35.5) |  |
| Having hypertension | No | 814 (81.2) | 189 (18.8) | <0.01 |
|  | Yes | 129 (46.1) | 151 (53.9) |  |
| Having diabetes | No | 888 (79.5) | 229 (20.5) | <0.01 |
|  | Yes | 55 (33.1) | 111 (66.9) |  |
| Having other neurological disease | No | 918 (73.9) | 324 (26.1) | 0.065 |
|  | Yes | 25 (61.0) | 16 (39.0) |  |
| Having family history of neurological disease | No | 873 (73.4) | 316 (26.6) | 0.825 |
|  | Yes | 70 (74.5) | 24 (25.5) |  |

The peripheral neuropathy was classified as "yes" for individuals with mild to severe peripheral neuropathy, while "no" referred to those with normal conditions.

Table 5 becomes Table 4 for predictors of peripheral neuropathy using multiple logistic regression (n=1283)

Comment 4: English editing is needed.

Answer: We have sent the manuscript for English editing as suggested and attached the certificate of proof reading.
